# Persistence of bloodstains on domestic surfaces treated with detergents: experimental evaluation using Combur Test^®^

**DOI:** 10.1007/s00414-026-03742-z

**Published:** 2026-02-18

**Authors:** Matteo Antonio Sacco, Valerio Riccardo Aquila, Roberto Raffaele, Saverio Gualtieri, Maria Cristina Verrina, Giuseppe Chiaravalloti, Isabella Aquila

**Affiliations:** 1 Institute of Legal Medicine, Department of Medical and Surgical Sciences, “Magna Graecia” University of Catanzaro, Catanzaro, 88100 Italy; 2 Department of Medical and Surgical Sciences, “Magna Graecia” University of Catanzaro, Catanzaro, 88100 Italy; 3 Nucleo Investigativo, Comando Provinciale dei Carabinieri, 88100 Catanzaro, Italy

## Abstract

The identification of latent blood traces on cleaned surfaces represents a crucial challenge in forensic practice, especially in cases of intentional removal attempts by the perpetrator. This study evaluated the effectiveness of Combur Test^®^ strips, based on the peroxidase activity of hemoglobin, in detecting residual blood traces on nine types of domestic surfaces subjected to cleaning with five different detergents, applied either immediately or after 48 h from deposition. Ninety samples were analyzed, classifying reactivity on a semi-quantitative scale (0–3) and subjecting the data to statistical analysis. Surface type proved to be the main discriminating factor (ANOVA p < 0.001): cardboard, synthetic leather, and cotton retained higher mean reactivity values (2.70–2.80), whereas plastic, stainless steel, and PVC showed lower values (1.3–1.5). Cleaning timing significantly influenced results (t-test p = 0.013), with a reduction of mean reactivity in samples treated after 48 h (1.98) compared to those cleaned immediately (2.40), suggesting that progressive dispersion and more difficult rehydration of residues reduce test sensitivity as the time interval increases. In contrast, detergent type did not produce statistically significant differences (p = 0.251). Overall, strong reactions accounted for 43% of observations, while absence of reactivity was rare (1%). In conclusion, Combur Test^®^ is confirmed as a rapid and cost-effective method for preliminary screening of latent blood traces. However, its sensitivity is influenced by both substrate nature and timing of the crime scene investigation, highlighting the need for prompt intervention and integration with confirmatory tests in cases of smooth surfaces, recent cleaning, or delayed sampling.

## Introduction

In the field of forensic sciences applied to criminal investigations, the identification, localization, and collection of biological traces represent a crucial step for the accurate reconstruction of event sequences and the assignment of responsibility. In particular, the detection of blood traces constitutes one of the most frequent investigative activities, as blood is a biological fluid that, due to its physicochemical characteristics, readily deposits on environmental surfaces and carries information of both genetic and dynamic-criminological nature. Its presence can provide decisive evidence on the spatial positioning of the victim and the aggressor, the modalities of violent contact, and subsequent manipulation phases of the crime scene [1].

However, the highly incriminating nature of blood and widespread awareness of its probative potential often lead perpetrators to carry out cleaning and concealment actions aimed at compromising the integrity of traces and reducing their macroscopic visibility [1–2]. Mechanical removal with cloths and sponges, repeated washing with domestic detergents, and the use of highly oxidative chemical compounds, such as sodium hypochlorite or ammonia, are recurring strategies that can diminish the likelihood of recovering microtraces and compromise subsequent collection and laboratory analysis phases [3]. The intentional alteration of surfaces, combined with the time elapsed between the criminal event and the crime scene inspection, increases the probability of reduced detectability below the LOD and interpretative uncertainties, thus posing a risk of altering the evidentiary framework.

The literature provides numerous studies dedicated to the sensitivity of detection techniques for latent blood traces, including chemiluminescence (Luminol, Bluestar^®^), forced fluorescence using fluorescein-based reagents, and immunochromatographic confirmatory tests for human hemoglobin [4–5]. Although these methods offer high analytical sensitivity, they have operational limitations related to cost, procedural complexity, the need for controlled environments, and the risk of false positives resulting from interactions with oxidizing substances and detergents [4–5]. In operational contexts, especially in domestic and rural settings, there is therefore a need for preliminary diagnostic tools that are rapid, portable, low-cost, and easily interpreted even by personnel without specialized training.

In this application scenario, the use of Combur Test^®^ is situated—a diagnostic system based on reactive strips impregnated with chromogenic substrates, exploiting the peroxidase capacity of hemoglobin and heme proteins to catalyze the oxidation of a colorimetric indicator, producing a visible color change to the naked eye. Originally developed for urinary analysis and detection of microscopic hematuria, Combur Test^®^ has progressively been tested as a preliminary screening tool for detecting blood traces on solid surfaces, with promising results in terms of speed and ease of use. Pilot studies have shown that this method, while having lower sensitivity than chemiluminescent systems, offers the advantage of high operational practicality and immediate reading, making it suitable as a first-line tool during on-site investigations [6–8].

Nonetheless, there is a lack of systematic experimental data in the literature regarding the ability of Combur Test^®^ to maintain adequate sensitivity in the presence of common environmental interferents such as domestic detergents. The presence of oxidizing agents and surfactants can potentially degrade hemoglobin or alter the chromogenic reaction, leading to reduced detectability below the limit of detection (LOD) and reducing test reliability. Furthermore, factors such as substrate type (smooth or porous surfaces), cleaning timing, and drying conditions may interact in complex ways, affecting the retention capacity of blood residues and subsequent detection by rehydration.

In the forensic context, presumptive blood tests must be interpreted by balancing analytical sensitivity and specificity. While high sensitivity allows the detection of minute traces, limited specificity may expose the investigator to cross-reactivity with non-blood oxidizing substances commonly present in domestic environments. Moreover, the absence of a visible chromogenic reaction should not automatically be interpreted as a true negative result. In many operational scenarios, blood may still be present but reduced to concentrations below the LOD of the test, particularly following cleaning procedures or environmental exposure. In addition, chemical inhibition mechanisms induced by detergents, surfactants, or oxidizing agents may interfere with the peroxidase-based reaction, attenuating color development without implying complete removal of the biological material. These aspects underline the importance of contextual interpretation of presumptive test results and the need for confirmatory analyses.

The present experimental study was designed to systematically assess the effectiveness of Combur Test^®^ in detecting latent blood traces deposited on different surface types and subjected to chemical cleaning, simulating a realistic scenario of intentional trace removal. The primary working hypothesis was that the chromogenic reactivity capacity would be significantly influenced by the porosity and microstructure of the surface, the chemical composition of the detergent, and the cleaning timing. The study was structured according to a multifactorial experimental design with stratification of independent variables and inferential statistical analysis of results.

The ultimate goal is to provide practical guidance for crime scene technicians and forensic experts to enhance the value of an affordable, readily available diagnostic tool, highlighting its limitations and potential in cases where the scene has been altered by cleaning attempts. The possibility of including Combur Test^®^ among first-line field tools could represent a strategic advantage during the initial stages of the investigation, especially in poorly equipped environments or emergency contexts.

## Materials and methods

### Preparation of experimental surfaces

The study was conducted using nine types of surfaces commonly found in domestic and work environments, selected for their structural and chemical heterogeneity: plastic, glazed ceramic, untreated raw wood, pure cotton fabric, glass, stainless steel, PVC linoleum, corrugated cardboard, and synthetic leather. Each surface was cut into square samples measuring 5 × 5 cm, prepared under sterile conditions and cleaned to remove any environmental contaminants or production residues. Each 5 × 5 cm substrate piece was used for a single experimental condition only. No multiple stains were applied to the same substrate fragment, and no spatial overlap occurred between samples, thereby excluding any potential cross-reactivity or reagent diffusion effects. The samples were then left to dry for 30 min at a controlled temperature of 22 ± 1 °C and relative humidity of 50 ± 5%, in an environment free of biological contamination. Prior to blood application, all substrates were preliminarily cleaned using sterile distilled water and allowed to air-dry completely, in order to remove manufacturing residues or environmental contaminants. This preliminary cleaning was performed uniformly on all samples before blood deposition and therefore did not interfere with subsequent blood detection.

### Application and short-term drying of blood

On each substrate, a drop of 0.1 ml of fresh human blood obtained from a healthy volunteer (with informed consent) was deposited. The blood was applied using a calibrated micropipette and left to dry for 60 min at room temperature. This procedure realistically simulated the presence of a recent but not visible blood trace in the superficial coagulation phase. In this study, the term “aging” refers exclusively to short-term drying and stabilization of the bloodstain rather than long-term environmental degradation, which was beyond the scope of the present experimental design.

### Simulation of cleaning with domestic detergents

After the drying phase, the samples were divided into two experimental subgroups according to the cleaning timing: the first group was treated immediately, while the second was treated 48 h after deposition of the trace. The choice of the 48-hour interval aimed to simulate scenarios where the attempt to remove traces occurs later, at a temporal distance from the event.

In both temporal conditions, the samples were washed with five different types of detergents: a commercial solution containing sodium hypochlorite (bleach), an aqueous solution containing ammonia, a universal multipurpose degreaser, a mixture of water and neutral soap, and an ecological solution of vinegar and baking soda. The quantity of detergent used was 5 ml per sample. The application was carried out using a disposable microfiber cloth, applied with uniform circular rubbing of the surface for 30 s to reproduce typical household cleaning action. After cleaning, each sample was rinsed with 10 ml of sterile distilled water and left to dry again for at least 60 min at room temperature before testing. Untreated bloodstains on each substrate type were included as internal controls and tested at the same time intervals, allowing comparison between cleaned and non-cleaned conditions.

### Application of Combur Test^®^ and reactivity classification

Once dried, the samples were tested for residual blood using Combur Test^®^ diagnostic strips manufactured by Accudoctor^®^. The test principle is based on the peroxidase activity of hemoglobin, which catalyzes a chromogenic reaction forming a colored complex visible to the naked eye. For each sample, a drop of approximately 50 µl of sterile distilled water was applied to the previously contaminated central area to rehydrate any residual blood. The reactive strip was then applied for a duration between 5 and 10 s. The color change was observed within 60 s of removal and was classified semi-quantitatively according to four intensity levels: absent, weak, moderate, and strong, corresponding respectively to numerical values 0, 1, 2, and 3.

### Statistical analysis

A data matrix was created including independent variables (surface type, detergent type, cleaning timing) and the dependent variable (reactivity score). Descriptive analyses (mean, standard deviation, minimum, and maximum) were calculated for each factor.

The normality of residual distributions was verified by Shapiro-Wilk tests, residual histograms, and calculation of skewness index. When this index was below 1 in absolute value, the distribution was considered approximately normal. For comparisons among surface, detergent, and temporal groups, Type III ANOVA for independent samples was applied, followed by Bonferroni correction for multiple post hoc comparisons. For cleaning timing, an independent samples t-test was performed. The significance level was set at *p* < 0.05. All analyses were conducted using R software version 4.3.0.

## Results

The analysis of the ninety samples tested revealed considerable variability in Combur Test^®^ reactivity, significantly correlated with surface type, detergent used, and cleaning timing. The semi-quantitative coding of chromogenic reaction intensity, assigning scores from 0 to 3 (absent, weak, moderate, strong), made it possible to obtain a data matrix suitable for statistical processing. Overall, strong reactions were observed in more than half of the cases (43.3%), while moderate reactions accounted for 33.3% and weak reactions for 22.3%. Only 1.1% of samples showed a completely absent reaction. The global mean reactivity was 2.19 with a standard deviation of 0.82, indicating a generally high level of blood trace persistence even in the presence of common detergents (Table 1).

**Table 1:** Data collection: Combur test experiment

Sample ID	Surface	Detergent	Timing Condition	Combur Reactivity (Absent/Weak/Moderate/Strong)
C001	Plastic	Bleach	Immediate	Weak
C002	Plastic	Bleach	Delayed	Strong
C003	Plastic	Ammonia	Immediate	Moderate
C004	Plastic	Ammonia	Delayed	Moderate
C005	Plastic	Degreaser	Immediate	Weak
C006	Plastic	Degreaser	Delayed	Weak
C007	Plastic	Water and soap	Immediate	Moderate
C008	Plastic	Water and soap	Delayed	Weak
C009	Plastic	Vinegar + baking soda	Immediate	Moderate
C010	Plastic	Vinegar + baking soda	Delayed	Absent
C011	Glazed ceramic	Bleach	Immediate	Strong
C012	Glazed ceramic	Bleach	Delayed	Moderate
C013	Glazed ceramic	Ammonia	Immediate	Strong
C014	Glazed ceramic	Ammonia	Delayed	Moderate
C015	Glazed ceramic	Degreaser	Immediate	Strong
C016	Glazed ceramic	Degreaser	Delayed	Moderate
C017	Glazed ceramic	Water and soap	Immediate	Strong
C018	Glazed ceramic	Water and soap	Delayed	Weak
C019	Glazed ceramic	Vinegar + baking soda	Immediate	Moderate
C020	Glazed ceramic	Vinegar + baking soda	Delayed	Moderate
C021	Untreated wood	Bleach	Immediate	Moderate
C022	Untreated wood	Bleach	Delayed	Weak
C023	Untreated wood	Ammonia	Immediate	Strong
C024	Untreated wood	Ammonia	Delayed	Strong
C025	Untreated wood	Degreaser	Immediate	Moderate
C026	Untreated wood	Degreaser	Delayed	Weak
C027	Untreated wood	Water and soap	Immediate	Strong
C028	Untreated wood	Water and soap	Delayed	Moderate
C029	Untreated wood	Vinegar + baking soda	Immediate	Moderate
C030	Untreated wood	Vinegar + baking soda	Delayed	Strong
C031	Cotton fabric	Bleach	Immediate	Strong
C032	Cotton fabric	Bleach	Delayed	Strong
C033	Cotton fabric	Ammonia	Immediate	Strong
C034	Cotton fabric	Ammonia	Delayed	Moderate
C035	Cotton fabric	Degreaser	Immediate	Moderate
C036	Cotton fabric	Degreaser	Delayed	Moderate
C037	Cotton fabric	Water and soap	Immediate	Strong
C038	Cotton fabric	Water and soap	Delayed	Strong
C039	Cotton fabric	Vinegar + baking soda	Immediate	Strong
C040	Cotton fabric	Vinegar + baking soda	Delayed	Strong
C041	Glass	Bleach	Immediate	Strong
C042	Glass	Bleach	Delayed	Strong
C043	Glass	Ammonia	Immediate	Strong
C044	Glass	Ammonia	Delayed	Strong
C045	Glass	Degreaser	Immediate	Strong
C046	Glass	Degreaser	Delayed	Moderate
C047	Glass	Water and soap	Immediate	Strong
C048	Glass	Water and soap	Delayed	Moderate
C049	Glass	Vinegar + baking soda	Immediate	Moderate
C050	Glass	Vinegar + baking soda	Delayed	Moderate
C051	Stainless steel	Bleach	Immediate	Weak
C052	Stainless steel	Bleach	Delayed	Moderate
C053	Stainless steel	Ammonia	Immediate	Moderate
C054	Stainless steel	Ammonia	Delayed	Weak
C055	Stainless steel	Degreaser	Immediate	Weak
C056	Stainless steel	Degreaser	Delayed	Weak
C057	Stainless steel	Water and soap	Immediate	Weak
C058	Stainless steel	Water and soap	Delayed	Moderate
C059	Stainless steel	Vinegar + baking soda	Immediate	Strong
C060	Stainless steel	Vinegar + baking soda	Delayed	Weak
C061	PVC/Linoleum	Bleach	Immediate	Weak
C062	PVC/Linoleum	Bleach	Delayed	Weak
C063	PVC/Linoleum	Ammonia	Immediate	Moderate
C064	PVC/Linoleum	Ammonia	Delayed	Weak
C065	PVC/Linoleum	Degreaser	Immediate	Weak
C066	PVC/Linoleum	Degreaser	Delayed	Weak
C067	PVC/Linoleum	Water and soap	Immediate	Moderate
C068	PVC/Linoleum	Water and soap	Delayed	Weak
C069	PVC/Linoleum	Vinegar + baking soda	Immediate	Moderate
C070	PVC/Linoleum	Vinegar + baking soda	Delayed	Weak
C071	Cardboard	Bleach	Immediate	Strong
C072	Cardboard	Bleach	Delayed	Strong
C073	Cardboard	Ammonia	Immediate	Strong
C074	Cardboard	Ammonia	Delayed	Strong
C075	Cardboard	Degreaser	Immediate	Strong
C076	Cardboard	Degreaser	Delayed	Moderate
C077	Cardboard	Water and soap	Immediate	Strong
C078	Cardboard	Water and soap	Delayed	Strong
C079	Cardboard	Vinegar + baking soda	Immediate	Strong
C080	Cardboard	Vinegar + baking soda	Delayed	Moderate
C081	Synthetic leather	Bleach	Immediate	Strong
C082	Synthetic leather	Bleach	Delayed	Moderate
C083	Synthetic leather	Ammonia	Immediate	Strong
C084	Synthetic leather	Ammonia	Delayed	Strong
C085	Synthetic leather	Degreaser	Immediate	Strong
C086	Synthetic leather	Degreaser	Delayed	Moderate
C087	Synthetic leather	Water and soap	Immediate	Strong
C088	Synthetic leather	Water and soap	Delayed	Strong
C089	Synthetic leather	Vinegar + baking soda	Immediate	Strong
C090	Synthetic leather	Vinegar + baking soda	Delayed	Strong

**Fig. 1:**
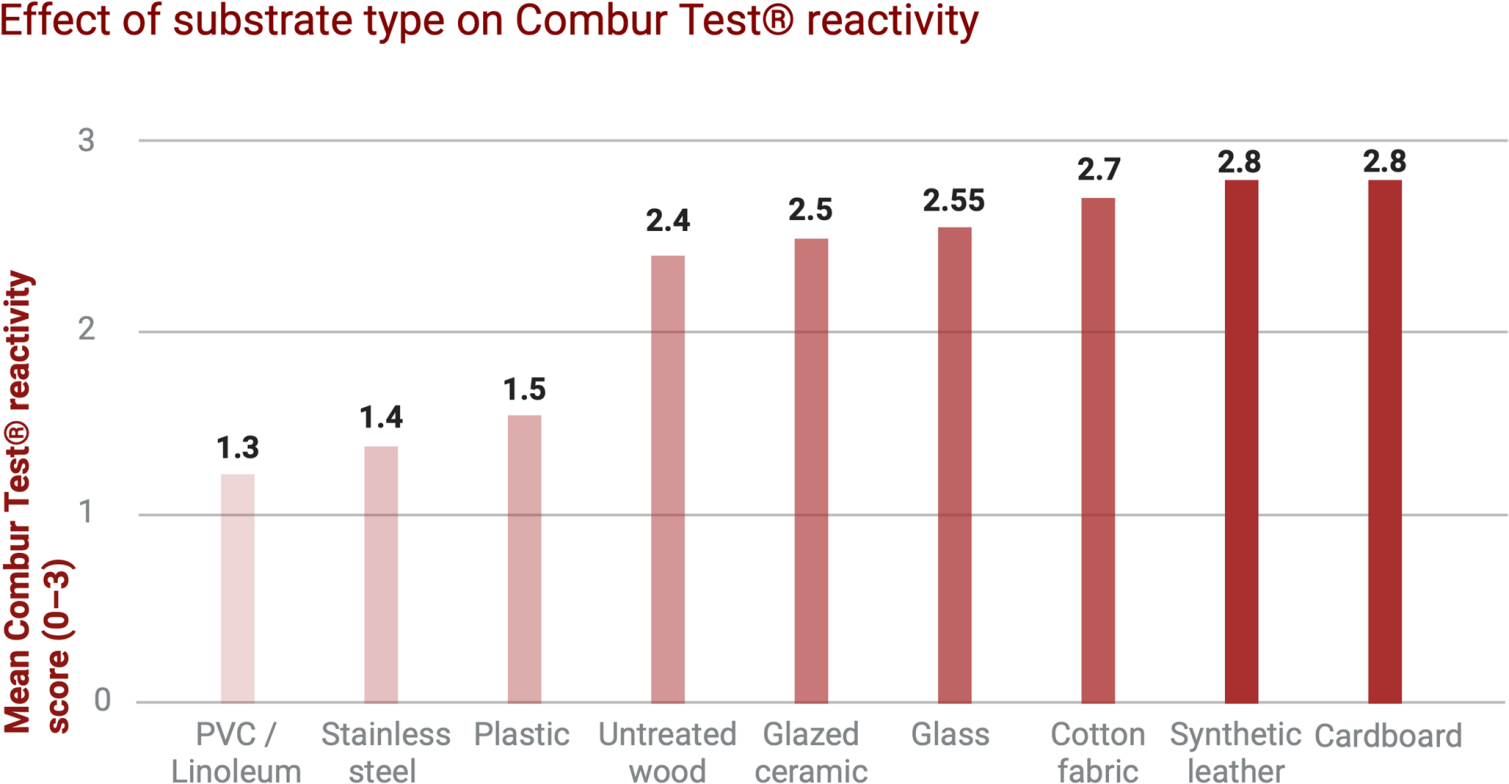
Mean Combur Test^®^ reactivity scores (0–3) across different substrate types after cleaning procedures

Stratification of results by deposition surface showed relevant differences. Materials characterized by marked porosity or fibrous structure—such as cardboard, synthetic leather, cotton fabric, and, to some extent, glass—showed the highest mean reactivity values (ranging from 2.55 to 2.85). In particular, cardboard had a mean of 2.80 (SD 0.42), with almost all cases showing a strong reaction. Synthetic leather had similar values (mean 2.80; SD 0.42), confirming the tendency of these substrates to retain blood residues even after cleaning procedures. Cotton fabric recorded a mean of 2.70, while glass, although being a smooth and non-absorbent material, showed a mean of 2.55, probably due to specific adhesion of dried blood to the surface. Conversely, smooth and hydrophobic surfaces such as PVC linoleum, stainless steel, and plastic showed significantly lower mean values (1.30–1.50), with a higher prevalence of weak or absent reactions. ANOVA confirmed the significance of these differences, yielding an F value of 9.7 and a p-value below 0.001 (Fig. 1).

Bonferroni post hoc analysis further identified significant differences in comparisons between cardboard and PVC, cardboard and plastic, cardboard and stainless steel, and synthetic leather and stainless steel. Additionally, significant differences were found between ceramic and PVC, stainless steel and cotton, stainless steel and glass, synthetic leather and PVC, plastic and cotton, plastic and glass, PVC and cotton, and PVC and glass (Table 2).

**Table 2:** Distribution of reactivity by surface

Surface	Absent (0)	Weak (1)	Moderate (2)	Strong (3)	Total
Cardboard	0	0	2	8	10
Glazed ceramic	0	1	5	4	10
Untreated wood	0	2	4	4	10
Metal (stainless steel)	0	6	3	1	10
Synthetic leather	0	0	2	8	10
Plastic	1	4	4	1	10
PVC / Linoleum	0	7	3	0	10
Cotton fabric	0	0	3	7	10
Glass	0	0	4	6	10
Total samples	**1**	**20**	**30**	**39**	**90**

The type of detergent used did not prove to be an additional discriminating factor, as no statistically significant differences were detected. Samples treated with bleach and ammonia showed mean reactivity of 2.20 and 2.40, respectively, and a lower frequency of weak or absent reactions. Conversely, neutral detergents and natural solutions such as water and soap and vinegar with baking soda showed similar mean reactivity (2.30 and 2.20, respectively), with strong reactions observed in approximately 43% of samples. The multipurpose degreaser yielded an intermediate mean of 1.8 (Table 3).

**Table 3:** Distribution of reactivity by detergent

Detergent	Absent (0)	Weak (1)	Moderate (2)	Strong (3)	Total
Vinegar + baking soda	1	2	8	7	18
Water and soap	0	4	5	9	18
Ammonia	0	2	6	10	18
Bleach	0	5	4	9	18
Degreaser	0	7	7	4	18
Total samples	**1**	**20**	**30**	**39**	**90**

Cleaning timing significantly influenced results. Immediate cleaning after deposition yielded a mean reactivity of 2.40 (SD 0.75), while delayed cleaning after 48 h showed a mean of 1.98 (SD 0.84). This difference was significant in the t-test (T = 2.5, *p* = 0.010). Cardboard subjected to delayed cleaning had a mean reactivity of 2.60, while the same surface cleaned immediately had a higher mean of 3.00. Plastic and stainless steel, conversely, exhibited lower mean scores (plastic: 1.6 immediate, 1.4 delayed; stainless steel: 1.6 immediate, 1.4 delayed) (Table 4).

**Table 4:** Distribution of reactivity by cleaning timing

Cleaning Timing	Absent (0)	Weak (1)	Moderate (2)	Strong (3)	Total
Immediate	0	7	13	25	45
Delayed (48 h)	1	13	17	14	45
Total	**1**	**20**	**30**	**39**	**90**

## Discussion

The results emerging from this study confirm that the detection of latent blood traces using Combur Test^®^ is strongly influenced by a complex interaction between the nature of the deposition substrate, the physicochemical characteristics of the detergents used, and the timing of the cleaning intervention. These findings align with the extensively documented forensic literature highlighting the vulnerability of blood traces to physicochemical degradation processes and the importance of a rigorous methodological approach for their identification.

Surface type emerged as the main determinant of residual reactivity, with statistically significant differences confirmed by Type III variance analysis (*p* < 0.001). Porous and micro-absorbent substrates such as cardboard, cotton fabric, and synthetic leather exhibited a marked tendency to retain blood traces even under experimental conditions of repeated cleaning and after a prolonged interval from deposition [9]. This behavior is attributable to the intrinsic capacity of such materials to absorb the biological fluid by capillarity and promote protein anchoring within the fibrous matrix. Progressive coagulation and evaporation of the carrier water lead to the formation of a stabilized protein network that hinders mechanical removal and reduces the degradative effectiveness of chemical agents. These dynamics were confirmed by the highest mean reactivity values observed for cardboard (2.80), synthetic leather (2.80), and cotton fabric (2.70), which maintained a prevalence of strong reactions even after cleaning.

The behavior of smooth, hydrophobic surfaces (plastic, stainless steel, PVC/linoleum) was consistent with the working hypothesis. These materials demonstrated a lower capacity to retain residual blood and greater susceptibility to removal by rubbing and detergent action. The observed mean reactivity values (1.3–1.5) and the prevalence of weak or absent reactions reflect the combination of factors: low initial adhesion of fresh blood, reduced retention of protein residues, and higher homogeneity of the surface, which limits microspaces for anchoring. However, it is noteworthy that in a non-negligible portion of samples, even smooth surfaces showed moderate or strong reactions. This finding highlights that micro-surface morphology, possible imperfections, and the heterogeneity of blood distribution can significantly affect results, generating a degree of intra-group variability that requires cautious interpretation.

A particularly interesting aspect was the behavior of glass, which exhibited intermediate mean reactivity values (2.6), higher than those of other smooth surfaces. This phenomenon could result from a combination of mechanical adhesion of the dry blood film, facilitated by the surface tension of glass, and the absence of background color interference during visual reading of the color change. Indeed, it is known that perception of the chromogenic reaction can be enhanced on reflective, uncolored surfaces, making the semi-quantitative classification of the color shift easier.

Regarding the influence of detergents, statistical analysis did not reveal significant differences between experimental groups. This result suggests that the chemical composition of common household detergents, while potentially exerting oxidative or surfactant effects, does not represent a decisive discriminating factor in compromising residual reactivity, unlike what is observed with professional high-concentration agents. Sodium hypochlorite-based bleach, while showing a slight reduction in mean reactivity (2.22), did not result in systematic suppression of detection capacity, confirming that the oxidizing action of free chlorine primarily affects the superficial component of the blood deposit and is ineffective in the deep microspaces of porous surfaces.

Cleaning timing proved to be a statistically significant discriminating factor (t = 2.51; *p* = 0.013). The observation of higher mean reactivity in samples cleaned immediately (2.40) compared to those treated after 48 h (1.98) apparently contrasts with the hypothesis of greater difficulty in removing coagulated blood. In reality, this result can be explained by considering the different rehydration capacities of the residual blood: in aged samples, molecular adhesion of the clot to the substrate and complete dehydration reduce the availability of peroxidase substrate at the time of strip application, resulting in attenuation of the chromogenic reaction. In samples cleaned immediately, conversely, a fraction of the blood remains partially moist, with greater availability of heme fractions for testing, even in the presence of detergents [10–11].

In light of these data, it is evident that crime scene inspection must be conducted as promptly as possible. Indeed, although aged blood traces may show greater stabilization of residues within the surface matrix, our study demonstrates that after 48 h there is, on average, a significant reduction in detectable reactivity, likely linked to progressive dispersion of heme components and more difficult rehydration [11]. This circumstance implies that delays in evidence collection activities increase the probability of reduced detectability below the LOD, particularly in environmental contexts subject to ventilation, thermo-hygrometric fluctuations, and repeated cleaning actions.

Weak or absent chromogenic reactions observed in this study should not be interpreted as true absence of blood. Rather, they likely reflect a reduction of detectable hemoglobin below the LOD of the Combur Test^®^, potentially exacerbated by chemical inhibition induced by detergents or by reduced rehydration efficiency of dried residues. This distinction is crucial in forensic interpretation, as biological material suitable for confirmatory testing may still be present despite low presumptive reactivity. Negative or weak presumptive test results should therefore be interpreted with caution and considered indicative of reduced detectability rather than definitive evidence of blood absence.

From an operational perspective, the results confirm that Combur Test^®^ is a valid, low-cost preliminary screening tool capable of supporting investigative orientation during on-site inspection. Its ease of use and rapid results make it particularly suitable in resource-limited settings. However, the heterogeneity of sensitivity, critical issues related to timing, and intra-group variability require a prudent interpretive approach [12–13]. In particular, in cases involving smooth surfaces or prompt cleaning interventions, evidence should always be supplemented by highly specific confirmatory methods, such as immunochromatographic tests for human hemoglobin and genetic analyses, to consolidate probative value [14–15].

Some limitations of the present study should be acknowledged. The experimental design simultaneously explored multiple variables, including substrate type, detergent composition, and cleaning timing, which may benefit from increased replication in future studies to further strengthen statistical robustness. Moreover, the temporal intervals investigated represent short-term persistence rather than long-term aging of bloodstains. Extended aging periods and repeated cleaning cycles may provide additional insight into long-term detectability dynamics and should be addressed in future investigations.

In light of the experimental evidence, it is recommended that operational protocols include precise instructions for the rapid initiation of collection procedures and the appropriate contextualization of results based on substrate characteristics, detergent type, and time elapsed since the event. Future studies could further explore the role of additional variables, such as surface granulometry, repeated cleaning cycles, and interactions with organic solvents, to more accurately define the limitations and potential applications of this methodology.
